# Supplementary material for: Exploring Gluconamide-Modified Silica Nanoparticles of Different Sizes as Effective Carriers for Antimicrobial Photodynamic Therapy
Source: Nanomaterials (Basel). 2024 Dec 11;14(24):1982. doi: 10.3390/nano14241982 (PMC11728795; doi:10.3390/nano14241982)
Supplement: Supplementary file 1 [file nanomaterials-14-01982-s001.zip › nanomaterials-3310849-supplementary.pdf]

Article

Exploring Gluconamide-Modified Silica Nanoparticles of Different Sizes as Effective Carriers for Antimicrobial Photodynamic Therapy

Ruth Prieto-Montero[1,*], Lucia Herrera[1], Maite Tejón[1], Andrea Albaya[1,2], Jose Luis Chiara[2], Mónica L. Fanarraga[3] and Virginia Martinez-Martinez[1,*]

[1] Departamento de Química Física, Universidad del País Vasco-EHU, Facultad de Ciencia y Tecnología, Apartado 644, 48080 Bilbao, Spain
[2] Instituto de Química Orgánica General (IQOG-CSIC), Juan de la Cierva 3, 28006 Madrid, Spain
[3] Grupo de Nanomedicina-IDIVAL, Universidad de Cantabria, Herrera Oria s/n, CP 39011 Santander, Spain
* Correspondence: ruth.prieto@ehu.eus and virginia.martinez@ehu.eus

Index

1.1 Synthesis of silica nanoparticles and their post-functionalization S1

1.2. Quantitative data of PS amount on silica NPs *S1*

1.3 Photophysical characterization of singlet oxygen by direct method S2

1.4 In vitro assays, irradiation protocol S2

1.5 Structural and Chemical characterization (TEM, XPS and FTIR) S3-S5

1.1 *Synthesis of silica nanoparticles and their post-functionalization*

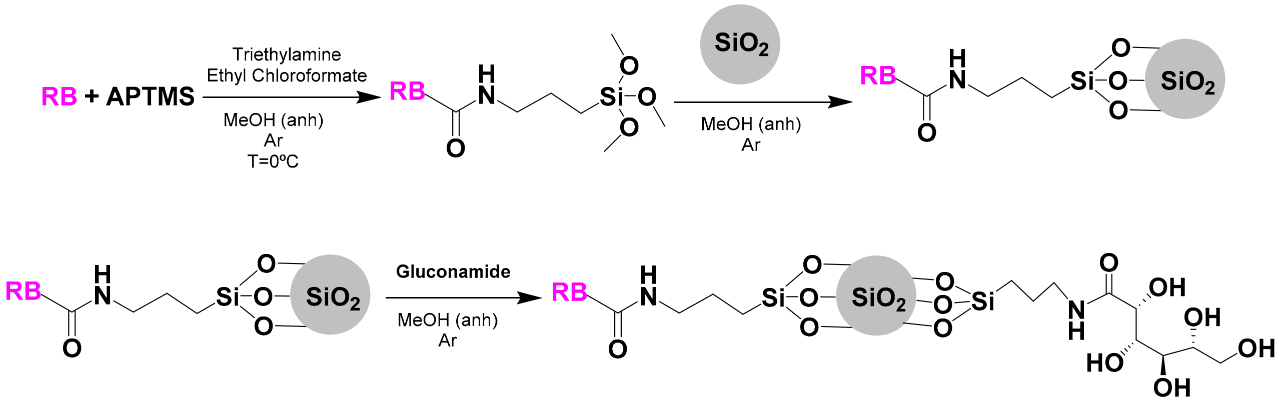

Scheme S1. Schematic representation of silica nanoparticles functionalization with RB and Gluconamide.

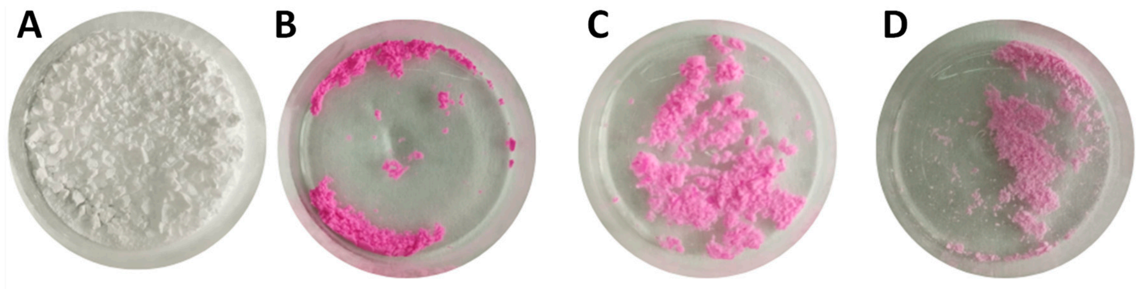

Figure S1. Images of original Silica nanoparticles (A), RB-G-20@SiNP (B), RB-G-80@SiNP (C), and RB-G-250@SiNP (D).

1.2. *Quantitative data of PS amount on silica nanoparticles*

Table S1. Surface RB density on silica nanoparticles of different sizes.

**sample**	**size in nm**	**Volume in nm³**	**Superficial area in nm²**	**RB umol per g NP**	**RB molecules per NP***	**RB molecules per nm²**
RB20@NP	21	4,800	1,385	7.0 ± 0.3	32 ± 2	0.023 ±0.0026
RB80@NP	80	270,000	20,106	2.1± 0.1	548 ± 27	0.027 ±0.0030
RB250@NP	260	9 200,000	212,371	0.8± 0.04	7228 ± 361	0.029 ±0.0033

*considering density of dense silica nanoparticles, ϱ = 1.6 g/mL (*Anal. Chem.* **2017**, *89*, 681–687)

1.3 *Photophysical characterization of singlet oxygen by direct method*

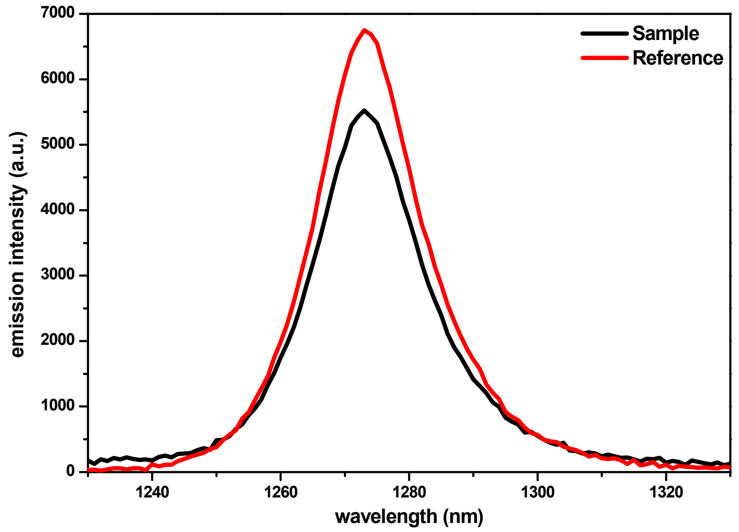

Figure S2. Representative singlet oxygen emission spectra for sample RB-G-25@SiNP (black) and reference Rose Bengal (red) excited at 550 nm.

1.4 *In vitro assays, irradiation protocol*

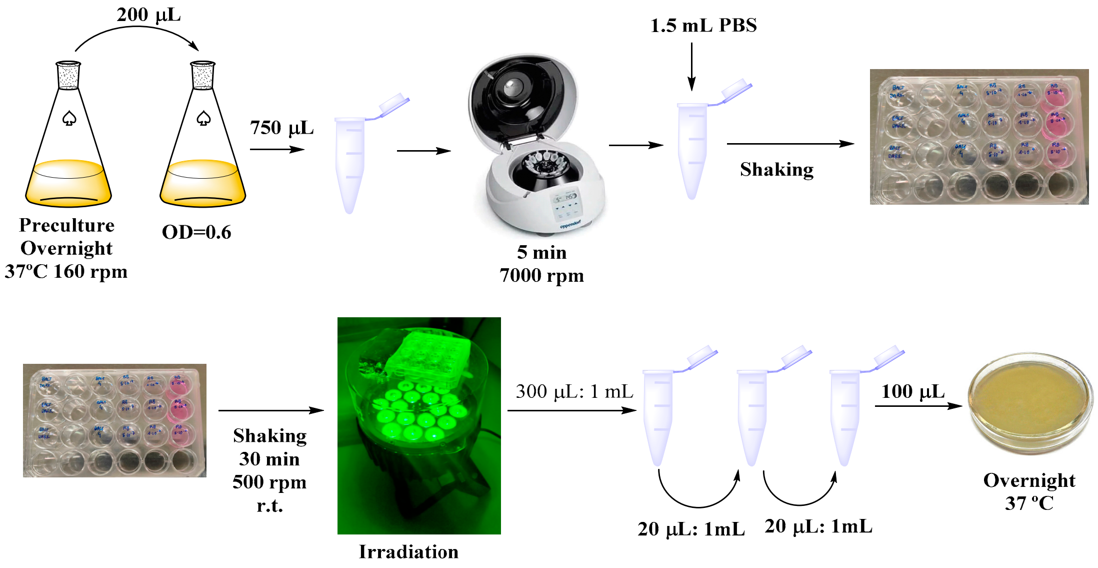

Scheme S2: Irradiation protocol for *E. coli* exposure.

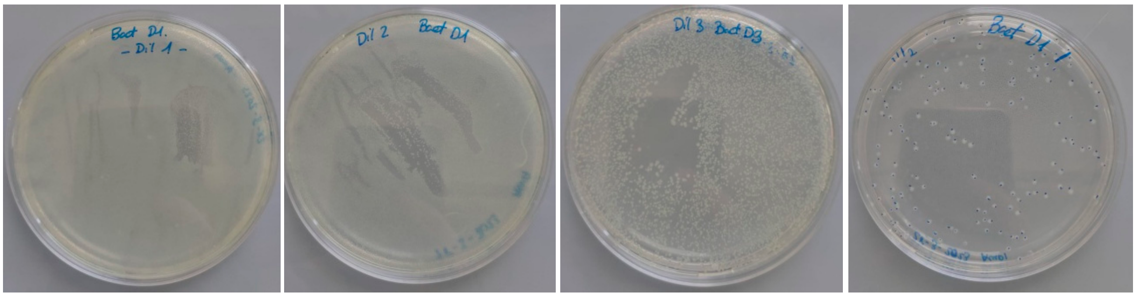

Figure S3. Overnight cultures of *E. coli* after serial dilutions for cell counting.

1.5 *Structural and Chemical Characterization*

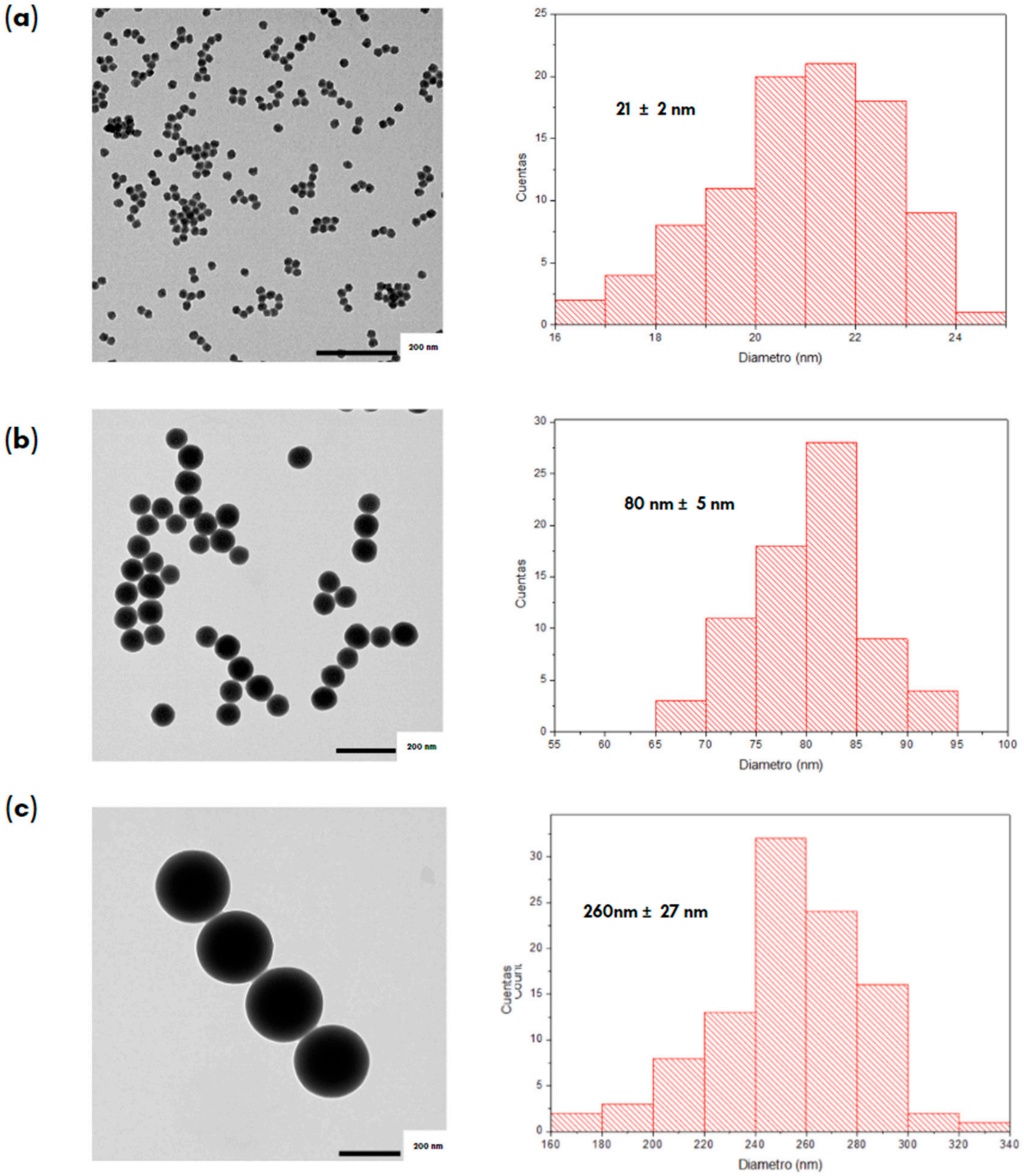

Figure S4: TEM images (left) and size distribution (right) of 20@SiNPs (a), 80@SiNPs (b), and 250@SiNPs (c) according to TEM images.

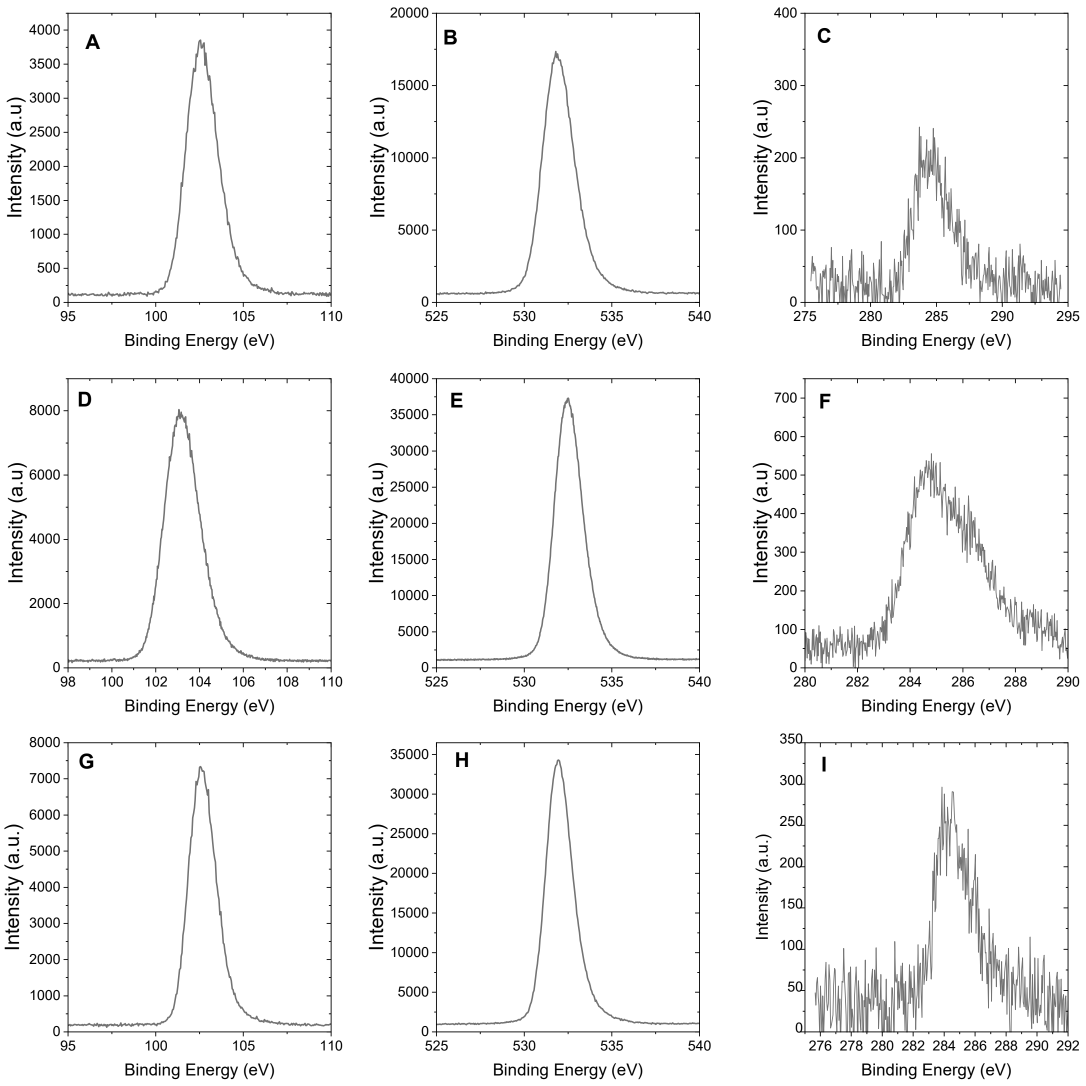

Figure S5. XPS spectra of Si 2p (left: A, D, G) O 1s (middle: B, E, H) and C 1s (right: C, F, I) for SiO_2 nanoparticles of 20nm (top: A, B, C), 80 nm (middle: D, E, F), and 250 nm (bottom: G, H, I).

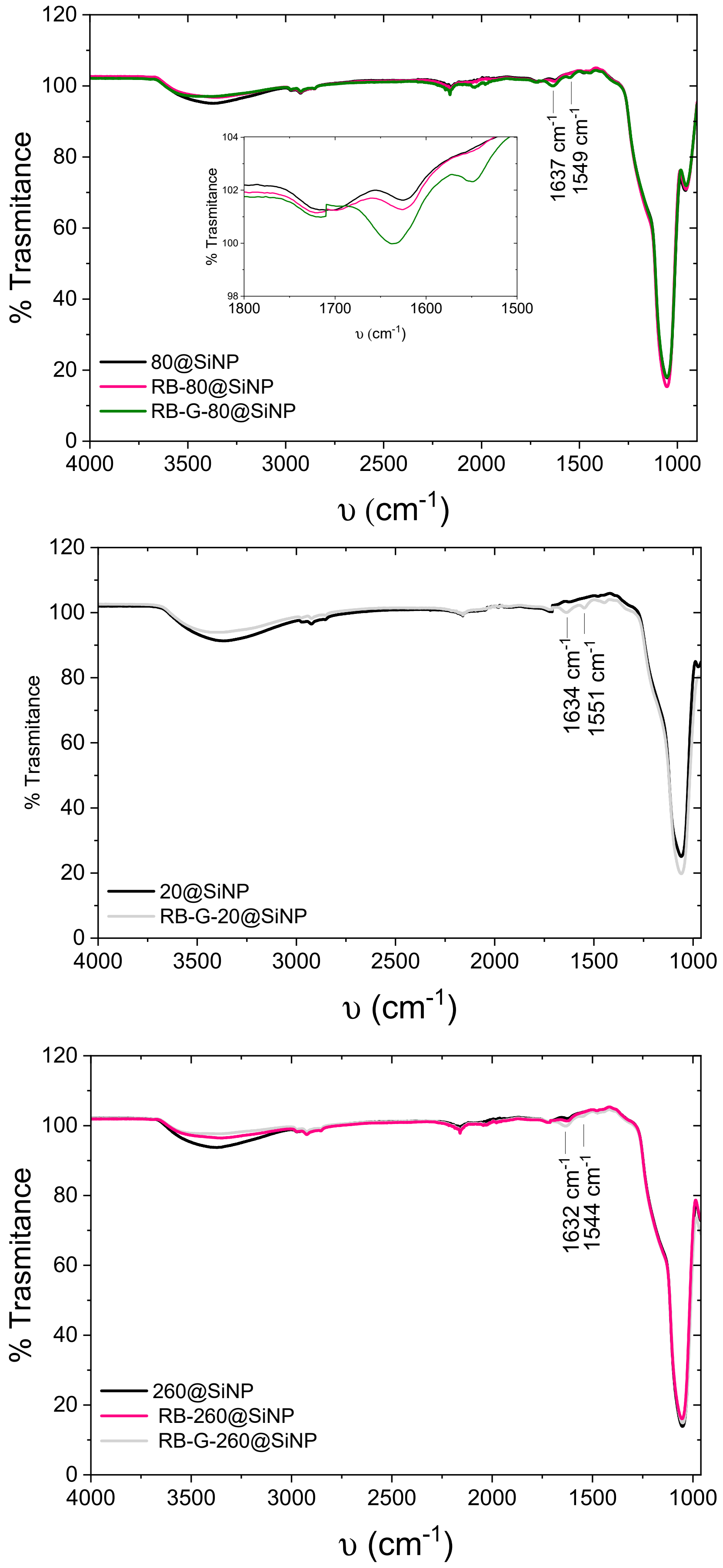

Figure S6: FTIR of bare and functionalized 80@SiNPs, 20@SiNPs and 250@SiNPs
